# Protein dynamics: The future is bright and complicated!

**DOI:** 10.1063/4.0000179

**Published:** 2023-02-27

**Authors:** Kwangho Nam, Magnus Wolf-Watz

**Affiliations:** 1 Department of Chemistry and Biochemistry, University of Texas at Arlington, Arlington, Texas 76019, USA; 2 Department of Chemistry, Umeå University, 901 87 Umeå, Sweden

## Abstract

Biological life depends on motion, and this manifests itself in proteins that display motion over a formidable range of time scales spanning from femtoseconds vibrations of atoms at enzymatic transition states, all the way to slow domain motions occurring on micro to milliseconds. An outstanding challenge in contemporary biophysics and structural biology is a quantitative understanding of the linkages among protein structure, dynamics, and function. These linkages are becoming increasingly explorable due to conceptual and methodological advances. In this Perspective article, we will point toward future directions of the field of protein dynamics with an emphasis on enzymes. Research questions in the field are becoming increasingly complex such as the mechanistic understanding of high-order interaction networks in allosteric signal propagation through a protein matrix, or the connection between local and collective motions. In analogy to the solution to the “protein folding problem,” we argue that the way forward to understanding these and other important questions lies in the successful integration of experiment and computation, while utilizing the present rapid expansion of sequence and structure space. Looking forward, the future is bright, and we are in a period where we are on the doorstep to, at least in part, comprehend the importance of dynamics for biological function.

## INTRODUCTION

Proteins represent the functional entity in the central dogma of molecular biology, and as such, they shape the function of all organisms.^1^ Although the structure-function paradigm states that three-dimensional structures of proteins define their functions,^2–4^ since the early days of induced fit^5^ and the MWC model for positive binding cooperativity,^6^ there has existed a firm understanding that dynamics^7^ is required for the function of proteins. There exists nothing as a completely static protein in nature, where the perhaps closest example of such are designed proteins like TOP7 from the Baker laboratory.^8^ Dynamics is manifested throughout the architecture of folded proteins, and even though the hydrophobic core of a protein is extremely densely packed, it has dynamic and fluid like properties as shown in a dynamically refined solution structural ensemble of ubiquitin.^9^ Here, we define protein dynamics as time dependent fluctuations of atomic coordinates,^10^ and these changes may be of reversible^11^ or irreversible nature.^12^ The timescale of dynamics is defined by the complexity of motion, where a more complex system with a larger number of atoms is less likely to make a concerted structural change, and hence, its corresponding dynamics is occurring on a slower timescale.^13^ For example, the timescale for vibration of atoms (that is relevant for the dynamics at transition states^13^) occurs on the fs timescale, whereas methyl-axis motions of side chains occur on the ps timescale.^14^ The dynamics of active site residues in an enzyme has been shown to occur on the ps-ns timescale,^15,16^ and domain movements in proteins generally occur on the *μ*s-ms timescale.^17^

Enzymatic catalysis is an area in biology where dynamics is intimately linked to function as all events in an enzymatic reaction cycle are dependent on fluctuations of atoms. This is evident for ligand binding and release,^17,18^ movement of catalytic side chains during the chemical transformation,^15^ and fast vibrations of atoms that are linked to the making and breaking of chemical bonds at the transition state.^13^ In this Perspective, we will focus on our view on future directions of understanding of protein dynamics with a special emphasis on enzymatic catalysis and the linkages between enzyme dynamics and function. Before discussing future directions, we elaborate briefly on “grand challenges” and the current state of the field. The perspective will be an attempt to provide a broad overview but will necessarily be influenced by our own efforts that have been centered around the metabolic enzyme adenylate kinase (AK) isolated from all three domains of life.^19^ Before focusing on enzyme dynamics, it is important to briefly mention other areas where protein dynamics is of fundamental importance for biological function and where future studies on dynamics surely will move the respective areas forward. Examples of these areas include (but are not limited to) allosteric signal propagation in receptors such as GPCR^20,21^ and receptor protein kinases;^22,23^ mechanisms underlying protein–protein interactions;^24,25^ the function of drug efflux by membrane bound transporters;^26^ the dynamic nature and mechanistic aspects of intrinsically disordered proteins (IDPs);^27–29^ motor proteins such as F1-ATPase, which is known to have an extreme efficiency in mechanochemical energy conversion;^30^ and dynamics of nucleic acids.^31,32^ Looking forward, there exist an abundance of biological functions that can only be fully understood at the molecular level by including quantitative approaches to protein dynamics.

## GRAND CHALLENGES IN BIOPHYSICS AND STRUCTURAL BIOLOGY

Although many of the fundamental aspects that conspire to lower the free-energy barriers in enzymatic catalysis are understood,^33^ we are still unable to predict enzyme function and mechanism directly from sequence and/or structural data alone. This challenge is likely to remain as one of the major goals in biophysics and structural biology during the foreseeable future.^34^ An even larger challenge is rational design of novel enzymes catalyzing reactions of biotechnological and commercial value. Although we cannot be sure exactly what information we are lacking to reach these goals, an obvious missing aspect is how the different dynamic modes of an enzyme contribute to generate the outstanding catalytic and regulatory properties of natural enzymes.^35^ Even though complicated, all the answers to enzyme function are there right in front of us, in which the obvious approach adopted by most researchers is to understand enzymatic catalysis by studying the enzymes that have evolved through natural selection. It is informative to compare the field of enzymology to how the field of protein folding has solved, at least in part, the “protein folding problem.” The field of protein folding^36^ was initially predominantly driven by experiment that uncovered many fundamental aspects such as the nucleation condensation mechanism,^37^ contact order dependency of protein folding kinetics,^38^ and the role of structural gate keeper residues.^39^ Following these and many other findings, the final steps toward quantitative prediction of folding trajectories and prediction of three-dimensional structures have been driven by computational approaches. The folding trajectory of small protein domains can now be computed, and this is due to the development of accurate force fields (based on physical principles) and sufficiently efficient computer soft and hardware.^40^ Even for larger proteins, prediction of their structures from sequence can also be done in a semi-rational manner that includes sequence analysis and machine learning with approaches like Alphafold2^41,42^ or RoseTTAfold.^43^ With these successes, it now becomes inevitable that the field of quantitative prediction of enzyme function and design will rely on massive contributions from computation.

Compared to the protein folding problem that, in principle, is sufficiently described by pairwise residue information, allosteric networks and propagated dynamical modes in enzymes are dependent on higher order interaction networks^34^ and, therefore, pose a significantly more complex problem. Enzymatic catalysis is further dependent on the interconversion between microscopic structural states that are only separated with on the order of RT (i.e., a few 2.5 kJ mol^−1^).^44^ Both the high-order dimensionality of interaction networks and the fine balance between structural states pose grand challenges to both our understanding of catalysis and the computational approaches. There has been some limited but encouraging success in rational enzyme design based on both computational design and following directed evolution with retro-aldolase activity^45,46^ and the use of resurrected ancestral enzymes as scaffolds for Kemp elimination activity.^47^ In these examples, the chemistry is relatively simple, i.e., relatively straightforward elimination reactions, but, nonetheless, catalytic efficiencies with *k*_cat_/*k*_uncat_ of 1.7 × 10^9^ have been reported.^46^ These examples demonstrate that there is potential for the rational enzyme design with high catalytic efficiency, but the step to catalysis of novel reactions including condensation reactions is still formidable. Another important aspect to consider in design is the modulation of ligand binding affinities. In many enzymes, such as GTPases and F1-ATPase, product release is rate-limiting and often accompanies large change of protein conformation. Although not a significant concern for small ligands, the binding affinities of larger, charged substrates and their on/off rates of binding are generally much more challenging to predict and, therefore, to incorporate in design efforts.

On the other hand, it is much easier to interfere with function rather than to create new functions and/or increase activity. To this end, there exist several examples of significant developments in inhibitor design based on, for instance, kinetic isotope effects (KIE) targeting transition state structures,^13^ de novo design of inhibitors against the SARS-CoV-2 spike protein,^48^ and virtual screening approaches.^49^ In order to successfully address the mentioned “grand challenges,” an inevitable step forward is the quantitative and mechanistic understanding of dynamics, an undertaking that (hopefully) can provide predictive power.

## PROTEIN DYNAMICS; STATE OF THE FIELD

Over the past decades, methodological and conceptual developments have led to considerable new discoveries that have subsequently contributed to the understanding of protein dynamics. However, many of these findings are phenomenological in nature and still await implementation into the quantitative understanding of enzymatic catalysis and design. In this section, we will highlight a selection of the latest findings that are relevant to protein and enzyme dynamics.

The physical principles that control dynamics are the underlying thermodynamic and kinetic properties of the systems including the protein, ligands, solvent, co-factors, salts, and any additional co-solutes. A fundamental difficulty in the quantitative understanding of dynamics is a dissection of thermodynamics into its enthalpic and entropic components and attributing them to the different constituents of the system (protein, ligand, etc.). A quantitative approach to deconvolute the residue-specific entropic contribution to the overall thermodynamics associated with ligand binding has been developed from NMR spin relaxation measurements for Galectin-3.^50^ This development led to the unexpected discovery that the ligand-bound state displays increased motion on the ps-ns timescale with a consequent increase in entropy. Additionally, allosteric binding to ligands can be driven by an increase in fast timescale motion as shown for the dynamically driven negative binding cooperativity of the catabolic activator protein.^51^ The mechanisms of ligand binding to enzymes have been the subject of intense research, and the induced fit^52^ and conformational selection^53^ models have attracted most attention. For example, from a combination of ancestral resurrection and quantitative biophysics, the selectivity of the anticancer drug Gleevec for Abl over Src kinase has been shown to depend on the magnitude of an induced fit conformational equilibrium.^54^ The conformational landscape of ligand free Abl kinase is intriguing, and, based on NMR spectroscopy, Abl has been shown to populate the major and active state at approximately 90% and two inactive states less than 5% for each.^55^ Interconversion between these states is of fundamental importance since the inhibitor imatinib has been shown to bind selectively to one of the inactive states of Abl.^55^ For insulin receptor kinase (IRK), we have recently shown that the free-energy difference between active and inactive conformations is very small, whereas the situation changes significantly for the kinase with phosphorylation of the so-called activation loop, i.e., for the active state kinase (Kwangho Nam manuscript under review). A similar mode of allostery has also been proposed for insulin-like growth factor 1 kinase (IGF-1RK).^56^ In a recent study from our laboratory, we observed that ligand dynamics (as observed from multiple crystallographic structures) also is a determinant for the broad substrate specificity of adenylate kinase isolated from the Asgard archaeal^57^ species *Odinarchaeota.*^19^

While the computational investigation of the ligand binding energetics, mechanisms, and their impacts on protein motions can be challenging, the energetics of the actual chemical step that reflects the activation barrier during the chemical event can be quantitatively approached with QM/MM approaches as shown for the enzyme adenylate kinase^58^ as well as many enzyme systems.^59–63^ A close connection between active site loop dynamics and the rate of catalysis for the two protein tyrosine phosphatases YopH and PTP1B has been observed from an NMR study in the Loria lab.^64^ In a study addressing side chain motions of active site residues, it was suggested that the transition state structure of the nucleoside monophosphate kinase UmpK is entropically restricted and surprisingly rigid.^15^ A linkage between barrier crossing and fs enzyme dynamics has been reported for human purine nucleoside phosphorylase and based on KIE.^65^

It is becoming increasingly apparent that the residues distant from the active site can influence the catalytic activity of enzymes.^66^ To this end, it was recently demonstrated that the motion of so-called evolutionary domains^67^ distant to the active site influences the activity of a protein tyrosine phosphatase.^68^ These observations suggest a concerted dynamics network that connects solvent exposed segments of the enzyme to the active site and provide an example of how higher order and dynamic interaction networks can affect enzymatic catalysis. Similarly, in RNase A, it has been shown that such long-distance effects can be achieved through the perturbation of intrinsic conformational dynamics of the enzyme.^69^

From a structural point of view, an emerging concept is to consider enzyme catalysis from an ensemble perspective. For example, an enzyme may adopt multiple conformational sub-states with differing reactivity toward bound substrates, and the most catalytically active state can be rare, high-energy states.^70^ Then, protein motions allow the exploration of the different conformational states on the ns–ms time scales while waiting for barrier crossing on the fs timescale.^71–73^ This notion has provided insight into the catalytic function of the enzyme ketosteroid isomerase.^74^ With the growing possibility of generating trajectories of proteins “in action” using time-resolved crystallography,^75^ this concept can now be tested for other enzymes. In the end of the day, enzymes have evolved over billions of years and developed the general and specialized dynamic properties required for their phenomenal rate enhancement.^35^ An example of these specialized properties is that an induced fit motion in members of the NMP kinase family of enzymes is controlled by a cation-π interaction between an arginine side chain and the base of the NTP substrate.^52,76^ Specialized properties may also co-evolve in different directions even for the same co-factor. For example (in addition to the general activation by Mg^2+^ in catalysis^16^), adenylate kinases from *E. coli* and *Odinarchaeota* respond in opposite directions in the presence of Mg^2+^ with respect to ligand binding. While Mg^2+^ accelerates conformational dynamics and ligand release in the *E. coli* enzyme by shifting an open/closed equilibrium in the direction of opening,^77^ the same ion drives the *Odinarchaeota* enzyme more toward the closed state.^19^ Thus, an observation of a specific phenomenon that appears clear and general can, instead, turn out to be a unique aspect for a specific enzyme (like the Mg^2+^ case just discussed). This possibility complicates the ability for us to draw general conclusions that can be widely used in, for instance, the design of novel enzymes.

In general, evolution solves an optimization problem in the most efficient way for a given functionality, and there exists an analogy for enzymatic catalysis to the fact that the evolution has optimized many different ways for achieving thermostability in hyperthermophilic proteins.^78^ The uniqueness of enzymes poses a fundamental challenge in search for general principles (or even more difficult one grand theory) that can provide molecular explanations to how dynamics contributes to catalysis. Therefore, a realistic path we can take now is to pursue the search for new functional and dynamic properties in the ever-expanding sequence and structural space of enzymes. From a methodological perspective, the initial understanding of how structural states are linked to function and dynamics was mainly inferred from stable ground sate structures. The methodology at hand, and also for this purpose, has traditionally been X-ray crystallography and complemented by NMR spectroscopy.^79^ Today, single particle cryo-EM has emerged as a second main technique for determining the structure of large proteins and macromolecular complexes.^80^ The latest addition to this toolbox is deep learning-based prediction of protein's ground state structures using Alphafold2^41,42^ and RosettaFold.^43^ It has been suggested, however, that these structure prediction programs are at their limit, and that the next logical step is to enable predictions of structural ensembles,^81^ a development that would massively open the door to large-scale approaches to understand protein dynamics. Currently, it is becoming increasingly important to characterize low populated high-energy states and other microscopic states transiently present during enzymatic functional cycles.^17,44^ Microscopic structural states can be quantified from (for instance) NMR spectroscopy using relaxation dispersion,^82^ paramagnetic relaxation enhancements,^83^ pseudo contact shifts,^72^ or residual dipolar couplings;^84^ X-ray crystallography using so-called pseudo-ensembles from data collected at room temperature;^74^ time-resolved X-ray solution scattering;^85,86^ single particle cryo-EM;^87^ and from time-resolved serial femtosecond crystallography.^88^ In summary, we are in an exciting period where complex questions related to dynamics are emerging together with an rapid expansion of sequence space and techniques for increasingly advanced characterization of microscopic structural states. The sections Challenges for the Future and Concluding Remarks describe a selection of challenges for the future in understanding the links between structure, dynamics, and function.

## CHALLENGES FOR THE FUTURE

The definition of “challenges for the future” is context-dependent, and we refrain from setting a goal such as integrating existing knowledge of protein dynamics into the design of novel enzymes. Instead, we argue that there still exist an abundance of unknown aspects of protein dynamics that need to be explored before such an ambitious goal can realistically be set. In the section below, we list a number of challenges that need to be addressed in both the short and longer term perspectives. These challenges are of necessity biased by our current understanding and perceptions of the field, and there are, of course, additional aspects to consider in the future.

## DYNAMIC INTERACTION NETWORKS, UNDERSTANDING BEYOND PAIRWISE INTERACTIONS

Transmission of information through proteins from allostery is a well-recognized and fundamental aspect of protein function rooted in the classic example of positive binding cooperativity for oxygen to hemoglobin, and the MWC formalism that accounts for this property^6^ through the dynamic equilibrium between “T” and “R” states. Identification of the underlying allosteric signal propagation networks through proteins can be made from statistical coupling analysis of coevolving residues applied to data from multiple sequence alignments^89^ as exemplified for TonB-dependent transporters^90^ and dihydrofolate reductase.^91^ From an experimental standpoint, an increasing number of studies have demonstrated the presence of allosteric networks that connect the dynamics of solvent exposed segments of proteins to the active site as discussed above for evolutionary domains.^68^ Such dynamic networks can be perturbed by single site variations as shown for the enzymes cyclophilin A^92^ and biliverdin reductase B^93^ and, therefore, highlighting their allosteric nature. Moving beyond the observation of these networks and generating explanatory models from fundamental principles are formidable tasks due to the complexity of the problem.

Unlike the structure prediction problem which can be tackled with pairwise interactions, allosteric networks are composed of clusters of interacting residues, and, hence, the underlying problem is much higher dimensional and complex. Moreover, the networks have evolved within the structural context and are embedded in surrounding layers or residues without significantly interfering with the intrinsic functions of the protein, e.g., catalytic activity (epistatic effects^54,94^). This complexity cannot be addressed with single site replacements, and the field needs to move beyond this (successful) paradigm and devise computational and experimental approaches that enable the deconvolution of higher order interaction networks. Some success in terms of quantitative understanding of linkages between dynamics and catalysis has been achieved from comparative approaches of mesophilic and hyperthermophilic enzymes.^18,19,95,96^ The strength of this approach is that the comparisons effectively remove the complication of background (epistatic) effects on functional dynamic networks. The dramatic expansion of sequence space for organisms of all domains of life has now been paralleled with a massive increase in structural models based on Alphfold2.^41^ These two developments will eventually allow comparative analysis using machine-learning methods to understand the functioning and evolution of allosteric networks. In order to address the dynamics in a “high-throughput” manner, necessary to respond to the increase in sequences and structural models, it is vital to develop computational methods capable of predicting dynamics accurately and efficiently. A big step in this direction would be the development of structural prediction methodology using data generated from MD simulations, so that they can predict high-energy (or microscopic) structural states, protein dynamics, and/or ensembles of protein structure under given experimental conditions.

Finally, in order to make the connection between dynamics and function, there is a need for significant development in fast and reliable way for accurate quantification of function (activity in the case of enzymes). Such developments can be both computational, such as through the development of new QM/MM methods, and experimental. From an experimental point of view, steps in this direction have been taken for enzymes with high-throughput production^97^ and quantification of catalytic parameters using microfluidic technology.^46,97^ Although these methods have significantly boosted throughput and enabled screening of thousands of variants, they may still not be sufficient to cover the higher order dimensionality of allosteric networks. Much larger throughput can be achieved with genetic methods such as phage^98^ or bacterial^99^ display or screening in yeast.^100^ The challenge with these methods is to design selective pressures that can probe for features of interest. In contrast, the development of new QM/MM methods has been relatively slow due to the computer-intensive nature of QM algorithms, while enzymatic functions require extensive sampling of enzyme conformations. However, in recent years, the development of machine learning approaches^101–105^ and large-scale high-performance computers utilizing both many-core CPU and GPU architectures has revived efforts to develop fast QM/MM algorithms for rapid prediction of enzyme activity. Overall, the field faces a formidable challenge, and the dissection and understanding of allosteric networks can only be accomplished with massive developments of high-throughput computational and experimental approaches. Eventually, these developments should be combined with protein structure predictions, to allow routine design of new enzymes.

## CONCERTED VAS RANDOM FLUCTUATIONS IN ACTIVE SITES

Although the chemical mechanisms of enzymes have been established for many enzymes, e.g., serine proteases^33^ and adenylate kinase,^16^ the underlying dynamic aspects of active site residues are currently not quantitatively understood. It has been proposed that the observed catalytic rate of an enzyme is the sum of the catalytic activity of all microscopic structural states that are populated.^34^ How are then these microscopic states kinetically connected? A fundamental, remaining question is whether the fast timescale dynamics of active site residues that is directly linked to catalysis and the population of microscopic structural states are of a random or concerted nature. A way forward to test this fundamental question is, for instance, by integrating computational structural biology (QM/MM and MD methods) with experiments to modulate the timescale and extent of the side chain dynamics that participates directly in the catalytic event. An illustrative example of how fast timescale dynamics can be quantified for catalytic side chains is a study centered on arginine side chains (N^ε^ atoms) in the enzyme monophosphate kinase UmpK.^15^

## CONNECTION BETWEEN LOCAL AND COLLECTIVE MOTIONS

Dynamics of enzymes has experimentally been quantitatively observed over a large dynamic range including slow *μ*s-ms motion (mostly connected to ligand binding and release)^106,107^ and fast ps-ns motion.^50^ An open question that remains underexplored is the possibility that dynamics on these vastly different time scales may be linked or coupled in some way. From a physics point of view, it is unlikely, if not impossible, that motions on distinct time scales are directly linked by a causal relationship. For this reason, most experiments have focused on exploring each timescale motion separately. However, there are two studies on adenylate kinase that approach the problem from different angles. The first study^108^ compared mesophilic and hyperthermophilic AK, and it was found that hinge regions, which are key to large-scale conformational transitions involved in the substrate binding/product release, displayed dynamics on the ps-ns timescale. At the same time, the large-scale collective conformational transition occurs on the slow *μ*s-ms timescale. These observations provide a correlation between the two different timescale dynamics, which can be interpreted as that fast timescale motions are required to enable the slower motion linked to substrate binding and release.^108^ In a more recent study from our lab, we propose that fast dynamics linked to the chemical step of AK is mechanistically linked to the slow collective opening/closing dynamics by virtue of the processes occurring on the same conformational trajectory.^16^ A similar connection has been observed from computation of human purine nucleoside phosphorylase.^109^ These studies are encouraging and indicate that the problem of dynamic connection between disparate time scales can be addressed through experiment and computation. For a complete understanding of enzymatic catalysis, these connections must be understood and the mechanistic level, such that they, in the long-term perspective, can be incorporated into enzyme design or optimization of existing enzymes.

## ACTIVE SITE DYNAMICS LINKED TO CHEMICAL MECHANISMS

Enzymes exhibit dynamic behavior, requiring exhaustive sampling of enzyme conformations for accurate calculations of enzyme mechanisms, barriers, and reaction free energies. The most significant contributor to the catalytic barrier is the dynamics of active site residues. QM/MM methods using MD simulations can be effective in capturing the effects of the dynamics of these residues that occur on the ps–ns timescale along the reaction trajectories. However, if the dynamics and orientations of active site residues are modulated by conformational motions on the μs-ms timescale, such as interconversion between different microstates, catalytic barriers must be determined based on these microstates. The effective barrier can then be calculated as an ensemble average of the catalytic barriers of all accessible microstates. Our recent studies^16^ and unpublished results on *E. coli* AK indicate that the catalytic residues of the enzyme adopt different sets of interaction networks along the catalytic reaction, which then couples to the different timescale collective protein motions and reaction energetics. This mode of connection does not occur via an instantaneous coupling between the two different timescale motions, but rather occurs at the mechanistic level to modulate the catalytic reaction and subsequent release of reaction products. While other examples of this type of coupling remain to be discovered, particularly in cases of allosteric enzymes such as in protein kinases, the challenge then is to identify these catalytically relevant conformational motions and explore their mechanisms through experiment and computations. To this end, a holistic approach is multi-level, multi-dimensional free-energy simulations that incorporate different levels of QM theories, all-atom and/or coarse-grand potentials, and enhanced sampling of local and global dynamics of the enzyme. However, such an approach, if it exists, is computationally very demanding, requiring significant development to achieve accuracy, efficiency, and flexibility in seamlessly integrating multiple levels of theory. Recently, various machine-learning approaches have been developed for enzymatic catalysis,^101–105^ but most of them are specific to each reaction step, lacking systematic generality and demonstrated transferability. The complexity is further increased when the enzymatic reaction involves multi-step processes, which is often the case; in such cases, multiple reaction mechanisms must be explored to identify a correct one, leading to a significant bottleneck in studying the mechanistic connection between catalytically relevant protein motions and enzyme design.

## MECHANISMS OF CONFORMATIONAL TRANSITIONS

Although a substantial attention has been directed toward the mechanism of ligand binding and primarily to the distinction between induced fit and conformational selection models, the detailed atomic fluctuations occurring on the pathway from free to substrate/ligand-bound states are less well understood. Understanding of the molecular mechanisms for such transitions may pave the way for development of inhibitors that bind selectively to the associated transition states. Currently, there exists some limited insight into how local unfolding and folding (so-called cracking) is associated with conformational changes.^110–112^ Another example is the finding that the transition state for binding of two intrinsically disordered proteins (IDPs) contains several native contacts that contribute to the stabilization of the protein complex.^113^ Further exploration of related mechanisms may, for instance, include MD simulations applied to transitions and single molecule FRET^114^ or other single molecule techniques such as optical tweezers.^115^

## DESIGN OF PLASTIC PROTEIN SCAFFOLDS

The field of protein design has undergone a remarkable development, and it is now possible (among many other aspects) to design cooperatively folding proteins,^116^ protein based inhibitors with high affinity,^48^ and (at least in principle) active sites.^117^ A remaining challenge is the design of plastic proteins that can transition between structural states in the absence of any external co-factors, for instance, by mimicking opening and closing dynamics observed in many enzymes^118^ and/or by introducing allosteric sites to enable allosteric regulation of enzyme activity. This is complicated, in part, by the fact that the energetic difference between the interconverting structural states in natural proteins can be as small as a few kJ mol^−1^, and that these small differences are difficult to rationally predict since they mechanistically depend on a few weak interactions or the release of a few ordered water molecules. Moreover, the entropic contribution to these small free-energy differences has not been rigorously incorporated into protein design efforts. Therefore, principles that will enable the design of plastic proteins would be a large step forward in the quantitative (or semi-rational) understanding of protein dynamics.

## IN SEARCH OF THE UNKNOWN

Proteins have evolved under specific selective pressures in order to perform a certain function under specific cellular and environmental conditions. Evolved functional properties may be of general or specialized character. For example, serine proteases contain catalytic triads,^33^ which are considered a general property, while *Odinarchaeota* AK has the unique ability to use all NTPs in the phosphorylation of AMP^19^ and is an example of a specialized property. In all cases, both the general and specialized properties are important for function. Above we have discussed some general properties linked to dynamics, and here, we highlight two approaches with significant potential for discovering new mechanistic insights into both function and dynamics. These approaches are bio-prospecting, i.e., the characterization of enzymes from organisms thriving in extreme habitats, and ancestral resurrection as a tool to back-date important molecular mechanisms. As an example of the potential for bio-prospecting our laboratory's work on AK isolated from *Odinarchaeota* (discovered at extremely hot habitats close to black smokers on the arctic mid-ocean ridge^119^) revealed a mechanism of broad substrate selectivity, a dependency of the active site on the enzyme's oligomerization states and a suggested model for enzymatic cold adaptation.^19^ Exploration of enzymes, for instance, from radiation tolerant bacteria such as *Deinococcus radiodurans*,^120^ from extreme habitats like Lake Bogoria in Kenya, which has an eco-system with a pH between 9.3 and 10.3 and a temperature close to 60 °C,^121^ salt-tolerant halobacteria,^122^ or from cold habitats like the Arctic Ocean^123^ has significant potential to reveal new general or specialized functions. Ancestral resurrection also provided significant insight into dynamics and function and was fundamental for, e.g., dissection of the mechanism underlying Gleevec selectivity to Src and Abl kinases,^54^ mechanisms of metamorphic proteins that can populate alternative structural ground states with different topology^124,125^ and insight into the mechanism of protein–protein association.^126^ However, these two directions have been dominated by the standard and time-consuming structure/function approach with a focus on a few selected enzymes, and therefore, the development of high-throughput approaches to dynamics and function will increase the potential of both bio-prospecting and ancestral resurrection. Both approaches have the unique advantage that enzymes from un-culturable organisms can be studied simply by expression of their respective gene products in suitable expression hosts. Both bio-prospecting and ancestral resurrection have been successful approaches so far, and with the large expansion of sequence space and increasing possibilities of accurate structural modeling, the potential of these approaches will continue to increase.

## CONCLUDING REMARKS

In this outlook article, we have identified a number of areas that we believe are important directions for the quantitative understanding of protein and, in particular, enzyme dynamics. The overview is necessarily biased by our own perception of the field, and there are certainly many other aspects of the dynamics that are not discussed and/or remain to be uncovered. Other areas of interest are, for instance, in-cell protein dynamics,^127–129^ identification of small molecule binders to intrinsically disordered proteins (IDPs) from structure and kinetics,^130^ development of structure determination methods to study protein allostery,^131^ and conceptual advances of the function of IDPs, for instance, in allostery.^132^ We conclude that although contemporary questions of protein dynamics are exceptionally complex, methodological advances and the dramatic increase in the availability of new sequences will undoubtedly push the field toward a quantitative understanding of the linkages between dynamics and function. The future is bright and complicated!

## Data Availability

The data that support the findings in this study are available within the article and the cited references.
